# Natural History of Cryptosporidiosis in a Longitudinal Study of Slum-Dwelling Bangladeshi Children: Association with Severe Malnutrition

**DOI:** 10.1371/journal.pntd.0004564

**Published:** 2016-05-04

**Authors:** Poonum S. Korpe, Rashidul Haque, Carol Gilchrist, Cristian Valencia, Feiyang Niu, Miao Lu, Jennie Z. Ma, Sarah E. Petri, Daniel Reichman, Mamun Kabir, Priya Duggal, William A. Petri

**Affiliations:** 1 Department of Epidemiology, Johns Hopkins School of Public Health, Baltimore, Maryland, United States of America; 2 International Centre for Diarrhoeal Disease Research, Bangladesh, Dhaka, Bangladesh; 3 Department of Medicine, Division of Infectious Diseases, University of Virginia, Charlottesville, Virginia, United States of America; 4 Department of Statistics, University of Virginia, Charlottesville, Virginia, United States of America; 5 Division of Biostatistics, Department of Public Health Sciences, University of Virginia, Charlottesville, Virginia, United States of America; 6 Department of Animal and Veterinary Sciences, Cummings School of Veterinary Medicine, Tufts University, North Grafton, Massachusetts, United States of America; 7 Emory University School of Medicine, Atlanta, Georgia, United States of America; Christian Medical College, INDIA

## Abstract

**Background:**

Cryptosporidiosis is a common cause of infectious diarrhea in young children worldwide, and is a significant contributor to under-five mortality. Current treatment options are limited in young children. In this study, we describe the natural history of *Cryptosporidium spp*. infection in a birth cohort of children in Bangladesh and evaluate for association with malnutrition.

**Methodology/Principal Findings:**

This is a longitudinal birth cohort study of 392 slum-dwelling Bangladeshi children followed over the first two years of life from 2008 to 2014. Children were monitored for diarrheal disease, and stool was tested for intestinal protozoa. Anthropometric measurements were taken at 3-month intervals. A subset of *Cryptosporidium* positive stools were genotyped for species and revealed that *C*. *hominis* was isolated from over 90% of samples. In the first two years of life, 77% of children experienced at least one infection with *Cryptosporidium spp*. Non-diarrheal infection (67%) was more common than diarrheal infection (6.3%) although 27% of children had both types of infection. Extreme poverty was associated with higher rates of infection (chi-square, 49.7% vs 33.3%, *p* = 0.006). Malnutrition was common in this cohort, 56% of children had stunted growth by age two. Children with *Cryptosporidium spp*. infection had a greater than 2-fold increased risk of severe stunting at age two compared to uninfected children (odds ratio 2.69, 95% CI 1.17, 6.15, *p* = 0.019) independent of sex, income, maternal body-mass index, maternal education and weight for age adjusted *z* (WAZ) score at birth.

**Conclusions/Significance:**

*Cryptosporidium* infection is common (77%) in this cohort of slum-dwelling Bangladeshi children, and both non-diarrheal and diarrheal infections are significantly associated with a child’s growth at 2 years of age.

## Author Summary

Diarrheal disease is a leading cause of death in young children worldwide. *Cryptosporidium species* are responsible for a large proportion of global burden of diarrhea. This study describes the natural history of cryptosporidiosis in a birth cohort of impoverished Bangladeshi children. Children were enrolled at birth and monitored for diarrhea twice a week for two years. Stool samples were tested for enteric protozoa. Children in this cohort had significant rates of malnutrition compared to the W.H.O. reference population, and extreme poverty was common. A majority of children were infected with *Cryptosporidium spp*, and we found that children who had at least one infection with *Cryptosporidium spp* during the two year follow up period were significantly more likely to have growth faltering by age 24 months. Cryptosporidiosis is a common infection in this cohort, and is associated with poverty and reduced growth during the first two years of life.

## Introduction

Diarrheal disease is the second leading cause of death in children under age five worldwide, with cryptosporidiosis estimated to be second only to rotavirus as the leading cause of moderate-to-severe diarrhea [1–3]. *Cryptosporidium spp*. are enteric protozoa, with 26 described species, but *C*. *hominis* and *C*. *parvum* most commonly infect humans [4,5]. Infection is characterized by profuse, watery diarrhea. Disease is self-limited in immune-competent adults, but can be associated with fulminant disease in immunocompromised patients and young children. *Cryptosporidium* infection has been associated with longer duration of diarrhea and 2–3 times higher mortality in young children [6,7].

Risk factors for cryptosporidiosis relate to the host, environment, and the species of the parasite. Immune-compromised adults, including those with HIV/AIDS or on immunosuppressive drugs, are at increased risk [8–10]. Young children, especially those with malnutrition, are more vulnerable, presumably due to lack of acquired immunity, however the biological mechanism is not clear [11–16]. In a child cohort in Bangladesh, almost 40% of children were infected with *Cryptosporidium* in the first year of life [15]. Breastfeeding and breast milk IgA have been identified as protective factors [15–17]. Host genetic susceptibility is also implicated as *Cryptosporidium* infection has been associated with *HLA* class II alleles and polymorphisms in the *mannose binding lectin* gene [18–19].

Environmental risk factors can be species specific, and include prolonged contact with domestic animals [20–22], overcrowded living conditions [23–26] and household contact with young children [12, 22, 27]. Sporadic epidemics have been reported in relation to contaminated water sources [28]. Livestock serve as an environmental reservoir for *C*. *parvum*, and transmission has been reported after direct contact with animals or drinking water contaminated by human or animal waste [20]. In contrast, humans are the only major reservoir for *C*. *hominis*, and transmission is related to person-to-person contact, thus urban settings and overcrowding have been associated with *C*. *hominis* [29]. In contrast to other enteric diseases, household income has not been reported to be a protective factor, and in one study was even associated with increased risk of infection [29,30]. Malnutrition has been identified as a risk factor for infection, and may potentiate adverse impact from infection [14]. Furthermore, studies from Brazil and Peru have noted short-term growth faltering after infection [31,32]. The relationship between cryptosporidiosis and malnutrition is complex and poorly understood.

In this study we describe the natural history of cryptosporidiosis in a peri-urban slum community near Dhaka, Bangladesh, over the first two years of life, a critical period for childhood growth and development [33]. Additionally, we identify risk factors in an endemic region, describe genetic diversity of the parasite, and test for contribution of infection on growth faltering in this population.

## Methods

The subjects studied were part of a community-based prospective cohort study of enteric infections in infants from a peri-urban slum of Mirpur, near Dhaka, Bangladesh from 2008 to 2014 [14,15]. Infants were enrolled at birth and actively monitored in their homes for diarrheal disease by twice-weekly visits of trained field research assistants. Study size was determined by estimates of diarrheal disease in this region and an estimated 10% annual loss to follow up [14]. Data on the subjects’ clinical symptoms were collected by interview of mothers. Diarrhea was defined as greater than three loose stools per day as reported by the mother.

Anthropometric measurements, consisting of length and weight, were taken once every three months. Each child was weighed on an electronic scale (Digital Baby & Toddler Scales, Seca 354). The length of the children was measured to the nearest centimeter (Infantometer Baby Board, Seca 416). Nutritional status was assessed by comparing weight and height with the weight and height of the World Health Organization (W.H.O.) reference population of the same age and sex, using W.H.O. Anthro software, version 3.0.1 [14].

Surveillance stool samples were collected from each subject once a month. Additionally, when diarrhea was reported by the mother, a diarrheal stool sample was collected. Both diarrheal and monthly stool samples were tested for *Cryptosporidium species* by real-time polymerase chain reaction. *Cryptosporidium spp*. infection was quantitated by qPCR, threshold cycle (C_t_). A *Cryptosporidium* diarrheal infection was defined by a child having a diarrheal stool positive with a negative preceding surveillance stool, and a *Cryptosporidium* asymptomatic or non-diarrheal infection was defined by a child having a surveillance stool test positive.

### Laboratory methods

All stool samples were tested for *Cryptosporidium species* by real-time polymerase chain reaction.

DNA Extraction was performed by a modified QiaAmp stool DNA extraction protocol which incorporates a three-minute bead-beating step to lyse *Cryptosporidium* oocysts (Qiagen, Valencia, CA) [34]. *Cryptosporidium* positive samples were detected using an assay previously described by our group that targets the *Cryptosporidium* Oocyst Wall Protein (COWP) [35]. COWP positive samples were further genotyped by the polymorphic region within the *gp60* gene using primers and conditions previously described [36,37] but with the modification that the amplifications were done using the MyFi (Bioline, Taunton, MA) with an activation 94°C for 5 min followed by 40 cycles in the primary PCR (94°C, 30 sec; 45°C, 55 sec; 72°C, 60 sec) with a final extension of 10min at 72°C. In the secondary PCR the cycling conditions [35] were (94°C, 30 sec; 55°C, 30 sec; 72°C, 30 sec) with a final extension of 10 min at 72°C.

Phusion high fidelity polymerase (ThermoFisher Scientific Inc, Waltham, MA) was used in a final PCR to add sequencing primer binding sites [38]. As necessary for the Phusion enzyme the activation step was at 98°C, 20 sec was followed by 34 cycles (98°C, 10 sec; 60°C, 20 sec; 72°C, 20 sec) with a final extension of 10min at 72°C. The QIAquick PCR purification kit was then used as per manufacturer’s instructions (Qiagen, Valencia, CA) to purify the amplicon. This was then sequenced by a contract research organization (GENEWIZ, South Plainfield, NJ) using standard protocols and the sequencing primers IS5 (AATGATACGGCGACCACCGA) or IS6 (CAAGCAGAAGACGGCATACGA).

The resulting sequences were then trimmed and aligned to the *gp60* reference sequence (Genbank Accession Numbers HQ631408, AY738187, AY738192, AY738184 and AF440638) using the Geneious Program (R7) (Biomatters, NZ). Consensus phylogeny was inferred from 500 bootstrap replicates to build a neighbor-joining consenus tree, and based on the Tamura-Nei model, the Nearest Neighbor method and the Geneious Program (R7). Branches that produced in fewer than 50% of the bootstrap phylogenies were collapsed.

### Statistical analysis

Differences in demographic factors between infected and uninfected children were assessed using two-sample t-test and chi-square according to exposure status (no *Cryptosporidium* infection, any type of *Cryptosporidium* infection, diarrheal *Cryptosporidium* infection, and exclusively non-diarrheal *Cryptosporidium* infection). Family monthly income (expressed in Bangladeshi Taka or BDT) below 6000 BDT/month was defined as “extreme poverty” based on the World Bank’s definition of less than 1.25 international dollars per person per day [39]. Anthropometric measures (height-for-age adjusted z-score or HAZ; weight-for-age adjusted z-score or WAZ; weight-for-height adjusted z-score or WHZ) were evaluated both as continuous and categorical variables. HAZ score at 24 months was used as the outcome in the final analyses evaluating nutrition, as HAZ is most representative of chronic malnutrition [40]. For all analyses evaluating malnutrition, we excluded children who fell into the bottom 2.3% of HAZ scores at birth (HAZ < -3.49), per WHO Global Database on Child Growth and Malnutrition guidelines [40]. Based on our cohort’s distribution of anthropometric indices, we classified children into four categories: 1) HAZ > -1; HAZ < = -1 and > -2 (mild stunting); HAZ < = -2 and >-3 (moderate stunting); and HAZ < = -3 (severe stunting).

To determine the time to first diarrheal and time to first asymptomatic *Cryptosporidium* infection, we performed Kaplan Meier survival analysis separately. All children who completed 24 months in the study were included, and those children without a *Cryptosporidium* infection within the first 24 months were censored at this time point. The probability of growth impairment at 24 months of age was evaluated using univariate and multivariable logistic regression with the categorized HAZ at 24 months as a polynomial response. Potential confounding variables including sex, family income, maternal body-mass index, maternal education, and WAZ at birth were adjusted in the multivariable analysis. Statistical significance was considered if *p* <0.05. Analyses were performed in Stata v.10 (Statacorp, USA). Comparison of *Cryptosporidium* quantitation in stool (threshold cycle) to severity of clinical infection was performed using the Kruskal Wallis test.

### Ethics statement

The study was approved by the Institutional Review Board of the University of Virginia and the Research and Ethical Review Committees of the International Centre for Diarrhoeal Disease Research, Bangladesh (icddr,b). Informed written consent was obtained from parents or guardians for the participation of their child in the study.

## Results

**Fig 1:**
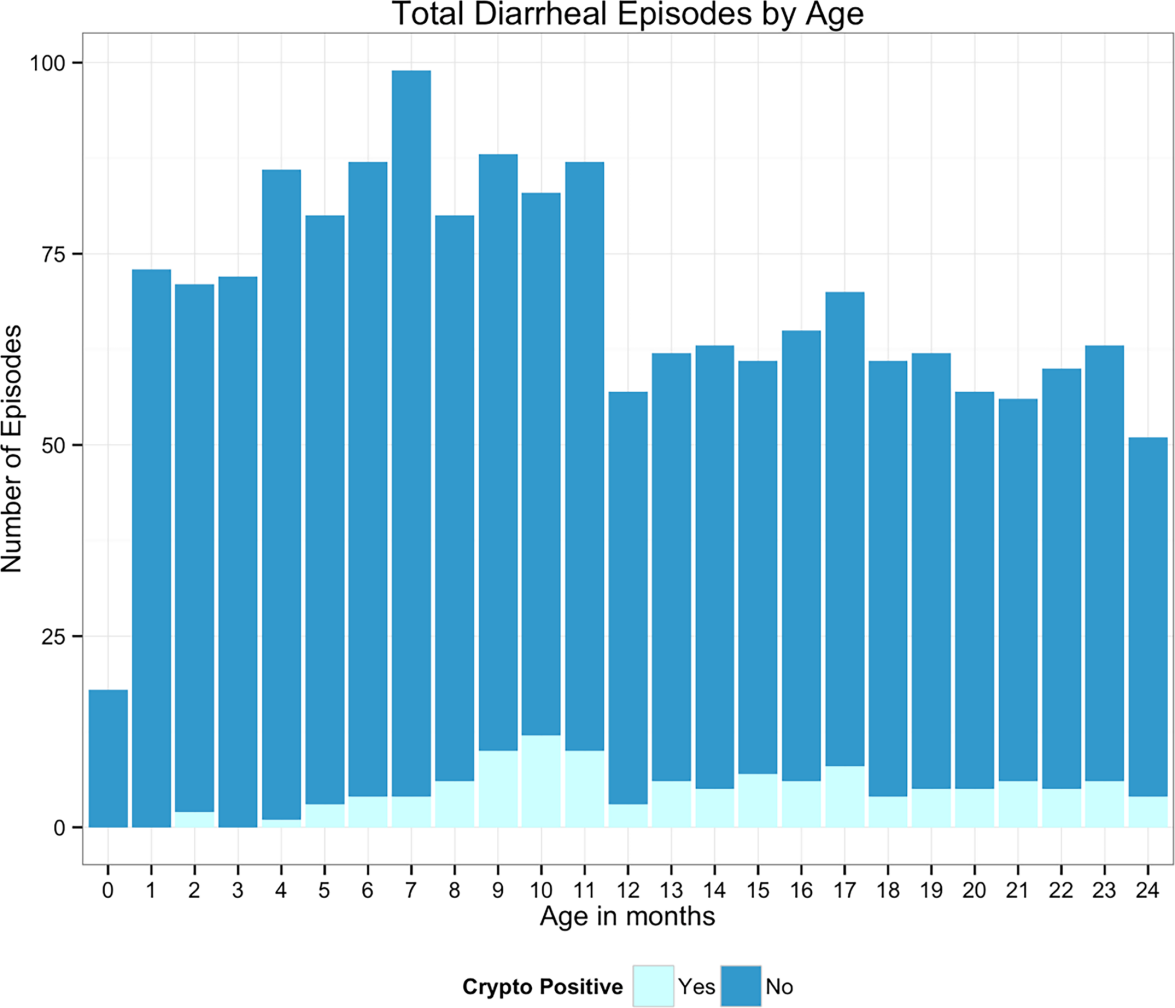
Total number of diarrheal episodes per month. The x-axis represents the child’s age in months, and the y-axis represents total number of diarrheal episodes per age-month. The dark blue segment represents number of total diarrheal episodes, and the light blue segment represents the proportion of diarrheal episodes per month that test positive for *Cryptosporidium species*. In each month, the total possible number of children included is 392. Cryptosporidium infection makes up a very small portion of the total burden of diarrhea in our cohort.

From 2008 to 2014, we followed 392 children from birth to 24 months of age. In this cohort, fifty-five percent of children were male. The median number of family members per household was 5, with range from 2 to 18. During the follow up period, there were 990 episodes of diarrhea in the first year of life (2.5 episodes of diarrhea/child), and 763 episodes in the second year of life (1.9 episodes of diarrhea/child). Over this time, 1712 diarrheal stools and 9231 surveillance stools were collected. In year one, 5.6% of all diarrheal episodes were positive for *Cryptosporidium spp*. and in year two 9.0% of diarrheal episodes were *Cryptosporidium spp*. positive (Fig 1). Mean age of onset for *Cryptosporidium* diarrhea was significantly greater than non-*Cryptosporidium* diarrhea (T-test, 13.9 months vs 11.3 months, p< 0.001). Forty-six percent of households were considered to be of “extreme poverty,” which is a higher rate than the Bangladesh national average of 31.5% [39]. Comparison of demographic factors between the 392 children who completed follow up with 237 children who were lost to follow up revealed no significant difference in gender (55% male vs 49%, chi square, p = 0.144), mean HAZ at birth (-0.96 sd 1.16 vs -0.9 sd 1.03, t-test, p = 0.513), and mean family income (6789.7 BDT sd 3049.0 vs 7092.4 BDT sd 4140, *t* test, *p* = 0.294).

**Fig 2:**
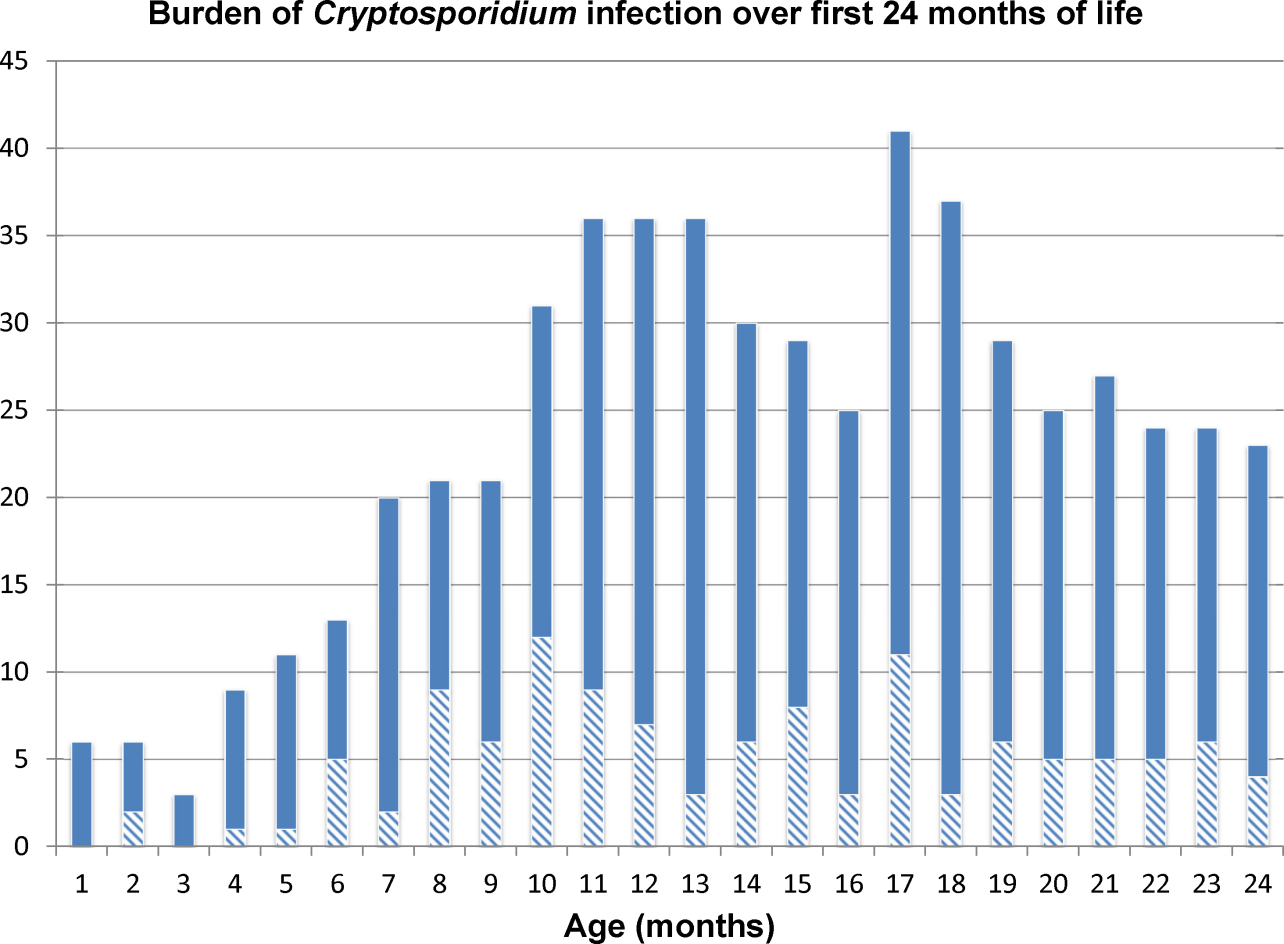
Total number of children with *Cryptosporidium* infection per month. The x-axis represents age of the child in months, and the y-axis represents total number of infected children per age-month. The solid-segment represents number of children with asymptomatic *Cryptosporidium* infection, and the lined-segment represents number of children with diarrhea from *Cryptosporidium* infection. In each month, the total possible number of children included is 392. A child who had both a diarrheal and asymptomatic stool positive in the same month was counted as having *Cryptosporidium* diarrhea. This figure demonstrates peaks of diarrheal *Cryptosporidium* infection at 10 and 17 months of age, and peaks of asymptomatic infection at 10–13 and 17 months of age.

By the end of the follow up period, 302 children (77.0%) experienced at least one infection associated with *Cryptosporidium spp* and of these, 100 (25.5%) children had at least one diarrheal infection and 283 (72.2%) children had at least one asymptomatic or non-diarrheal infection. A majority of *Cryptosporidium* detections were from non-diarrheal stools (Fig 2).

*Cryptosporidium spp*. infection was associated with a higher parasite burden in diarrheal disease, as *Cryptosporidium* positive diarrheal samples had lower CT values (median 33.35, interquartile range 6.8) than positive surveillance samples (median 35.50, interquartile range 5.6), (Kruskal Wallis, chi-square, *p* <0.0001). Of the 302 children with cryptosporidiosis during the two year follow up, 102 children had a second infection, 13 children had a third infection, and 3 children had 4 total episodes of cryptosporidiosis. There was no evidence of a decreasing parasite burden with repeated infection, as measured by threshold cycle (Wilcoxon test, paired between median C_t_ value of first vs second infection, 33.70 (IQR 6.6) vs 34.50 (IQR 7.2), *p* = 0.96; paired between median C_t_ value of second vs third infection, 34.50 (IQR 7.2) vs 35.40 (IQR 4.3) *p* = 0.62).

### Risk factors for *Cryptosporidium* infection

Table 1 summarizes demographic characteristics of children who became infected with *Cryptosporidium spp*. during the first 24 months of life. A higher proportion of *Cryptosporidium-*infected children came from extreme poverty (monthly income <6000 BDT/month) (*chi-square p* = 0.006). We found no association between increased risk of *Cryptosporidium* infection and HAZ at birth (*p* = 0.89), presence of an animal in the house (*chi-square*, *p* = 0.57), or family size (*chi-square*, *p* = 0.44).

**Table 1:** Comparison of demographics of 392 children who completed two years of follow up.

	No *Cryptosporidium* infection (n = 90)^a^	Any *Cryptosporidium* infection (n = 302^a^		Diarrheal *Cryptosporidium* infection (n = 100) ^a^		Non-diarrheal*Cryptosporidium* infection (n = 202)^a^	
			*p* ^*b*^		*p* ^*b*^		*p* ^*b*^
**Male %**	57	55	0.78	59	0.75	53	0.56
**Income < 6000 BDT %**	33	50	0.006	52	0.009	48.5	0.016
**Animal %**	6	7	0.57	7	0.68	7	0.56
**No Maternal education**	63	62	0.85	59	0.54	64	0.93
**Mean WAZ at birth**	-1.38	-1.41	0.83	-1.45	0.64	-1.39	0.96
**Mean HAZ at birth**	+/- 0.99	+/- 0.97		+/- 0.95		+/- 0.99	
	-0.94	-0.96	0.89	-1.02	0.60	-0.93	0.95
**Mean Maternal BMI**	+/- 1.15	+/- 1.16		+/- 1.00		+/- 1.23	
	21.7	21.2	0.21	21.4	0.52	21.2	0.150
**Exclusive breast feeding days**	+/-3.5	+/-3.2		+/-3.46		+/- 3.0	
	130.5	119.2	0.16	118.0	0.22	119.8	0.20
**Family size > 5%**	+/- 70.3	+/- 65.9		+- 70.5		+/- 63.7	
	42	38	0.44	37	0.86	36	0.32

^a^ Children having at least one *Cryptosporidium* infection (either in diarrheal or surveillance stool) over the first 24 months of life were included in the “Any *Cryptosporidium* infection” group, and all others were included in the “No *Cryptosporidium* infection” group. *Cryptosporidium* positive children were further divided into “Diarrheal *Cryptosporidium* infection”, which included all children who ever had a single diarrheal stool test positive for *Cryptosporidium*, and “Non-diarrheal *Cryptosporidium* infection” which included only children who had surveillance stools positive (children with both *Cryptosporidium* positive diarrhea and asymptomatic stools were excluded from this category). Each Cryptosporidium infection group was compared to the “No *Cryptosporidium* infection” group.

^b^ Chi-square or two-sample t-test

### Nutritional measures

**Fig 3:**
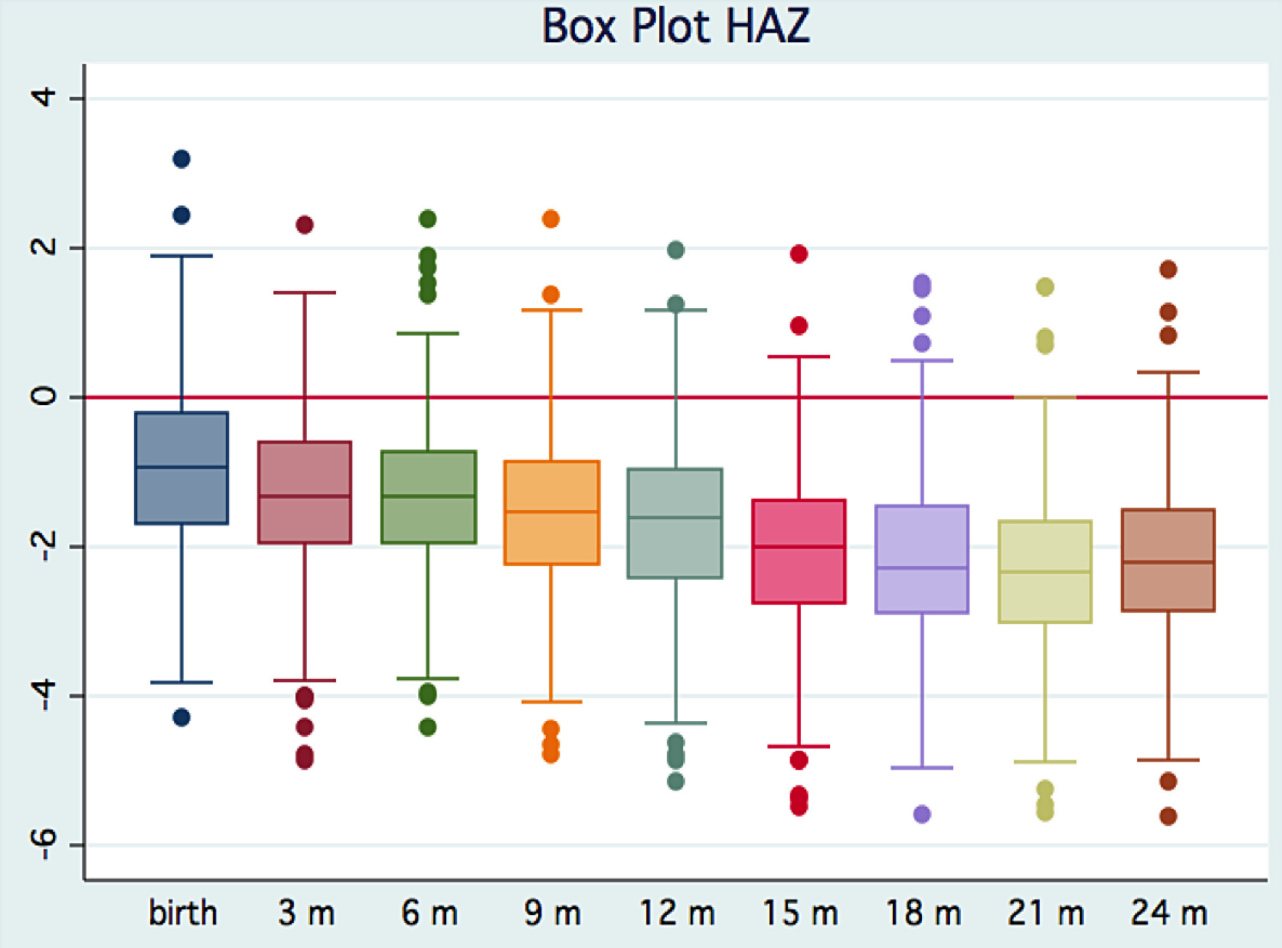
Box plot of mean height-for-age adjusted z-scores over first 24 months of life. In this cohort of 392 children, mean HAZ dropped continuously over the first two years of life, which was also consistently well below the W.H.O. reference population mean (indicated by red line).

Seventeen percent of children (68/392) met W.H.O. guidelines for moderate stunting (height-for-age adjusted z-score less than -2) at birth. By age two, 29.6%, 35.7%, and 21.1% of children met W.H.O. criteria for mild, moderate, and severe stunting, respectively. Over the first two years of life, the mean height-for-age adjusted z-score in this cohort fell consistently below the W.H.O. reference population. Fig 3 shows the steady decline in HAZ from birth to 24 months of age.

### Survival analysis for diarrheal and non-diarrheal infection

Fifty-percent of children had at least one non-diarrheal *Cryptosporidium* infection by 16 months of age, and 25% of children had a symptomatic infection by 2 years of age. The hazard ratio of an asymptomatic infection did not differ by sex (HR = 1.12, 95% CI 0.89. 1.42, p-value = 0.315) but was decreased for individuals with higher family income (HR = 0.74, 95% CI 0.58, 0.93, p-value = 0.011). The hazard ratio remained consistent for the symptomatic diarrheal infections.

**Fig 4:**
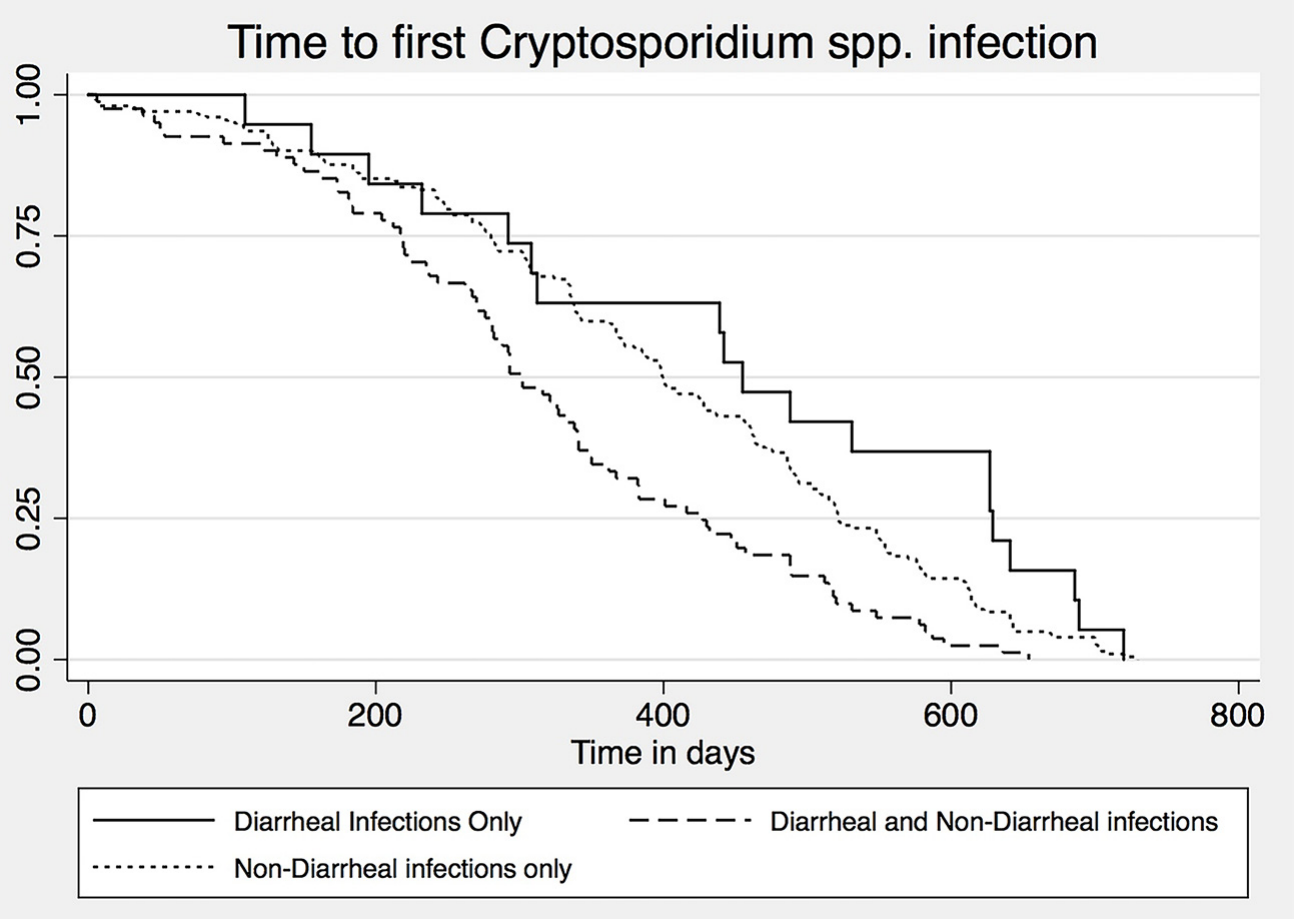
Kaplan Meier curve of the 302 children infected with *Cryptosporidium spp*. categorized by type of infection experienced (diarrheal, non-diarrheal, or both). There was no significant difference between timing of first asymptomatic versus symptomatic *Cryptosporidium* infection (HR 0.68, 95% CI 0.42, 1.09, *p* = 0.11). However, children who went on to develop both symptomatic and asymptomatic infection did get infected earlier in life (HR = 1.74, 95% CI 1.34, 2.27, *p* < 0.0001).

There was no significant difference in time to first asymptomatic or diarrheal infection (Fig 4). However, children who had both diarrheal and asymptomatic infections during the 24-month follow up period were infected at an earlier age (HR = 1.74, 95% CI 1.34, 2.27, p-value < 0.0001).

### *Cryptosporidium* genotypic diversity

**Fig 5:**
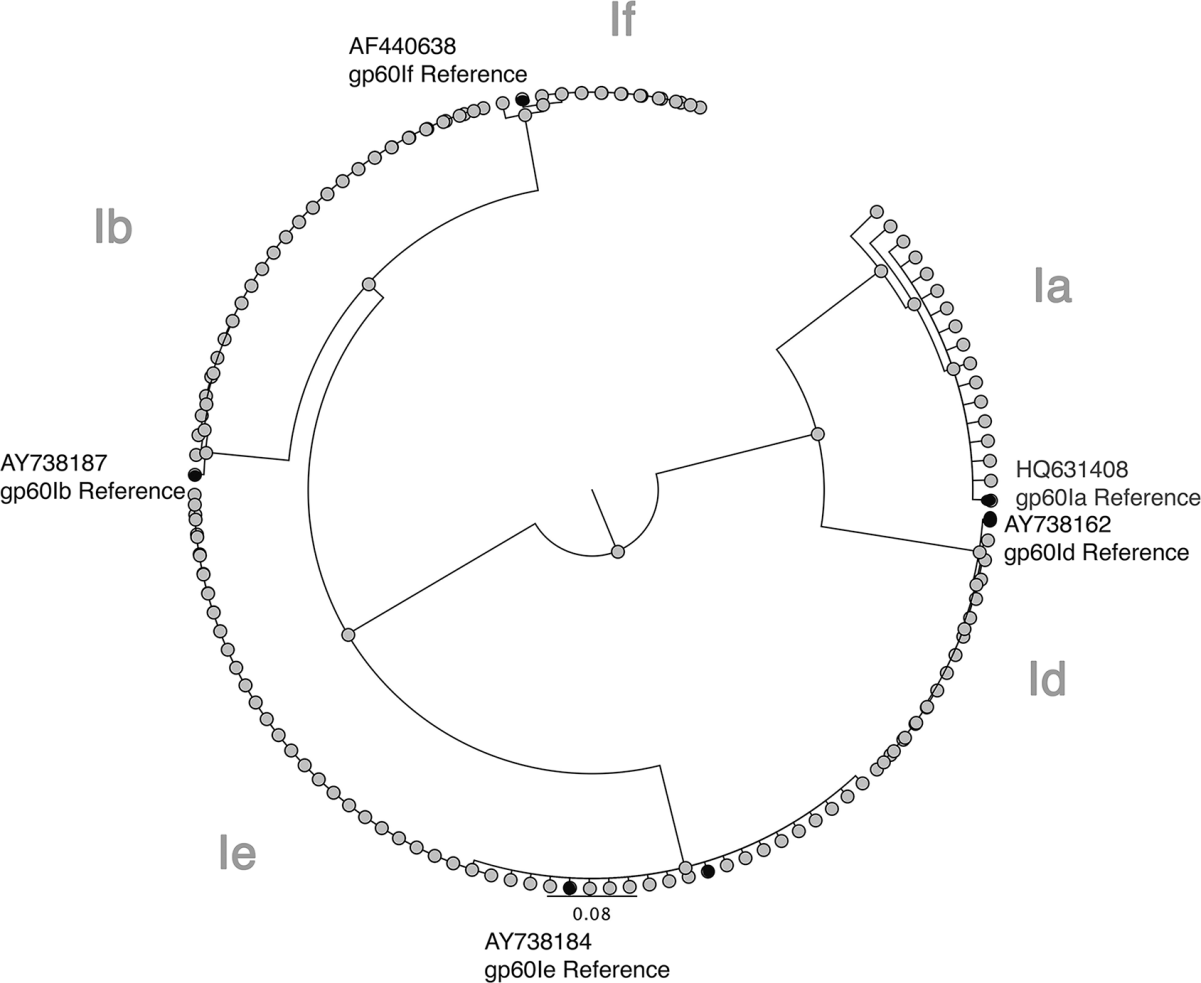
Phylogenetic Relationship of 101 *Cryptosporidium* parasites isolated in Bangladesh to each other (open circle) and to the reference sequences (closed circle) gp60Ia (HQ631408), gp60Ib (AY738187), gp60Id (AY738192), gp60Ie (AY738184), gp60If (AF440638). Neighbor-Joining consensus tree was drawn using the Geneious program (R7). Any apparent diversity is due to ambiguous bases.

A subset of diarrheal and surveillance stool samples (n = 238) testing positive for *Cryptosporidium spp*. were further typed by gp-60. *C*. *hominis* was the sole *Cryptosporidium species* in 92.4% of samples (n = 220) and *C*. *parvum* was the sole species identified in 3.4% (n = 8). In only five cases did we observe a mixed infection of *C*. *hominis* and *C*. *parvum*. *C*. *hominis* positive samples were further subtyped and the distribution of gp 60 alleles found in our study population was 1a (17.3%); 1b (21.4%); 1d (13.9%); 1e (40.5%); 1f (7.0%). Of these sequences, one hundred and one samples were of sufficient quality to subtype by multiple sequence alignment with GenBank reference sequences and phylogenetic analysis (Fig 5). In contrast to other molecular epidemiologic studies there was no *gp60* subtype diversity within our population (Ia, A14R1; Ib, A9G3R2; Id, A15G1; Ie, A11G3T3; If, A13G1) (32).

### Risk factors for growth faltering at 24 months

Using logistic regression, we observed that children with linear growth faltering at 24 months had a greater than 2-fold increased odds of experiencing any type of *Cryptosporidium spp*. infection during the first two years of life compared to non-stunted children (Table 2). The associated risk of *Cryptosporidium spp*. infection increased with the severity of stunting, and children with severe stunting at 24 months had a 2.69 times increased odds of *Cryptosporidium* infection (Table 2). The association of stunting and linear growth faltering at 24 months was present for both non-diarrheal “asymptomatic” and symptomatic *Cryptosporidium spp*. infections (Table 2). Stunting was associated with increased odds of infection even after adjusting for income, gender, maternal BMI, maternal education, days of exclusive breastfeeding, and nutritional status at birth. When we considered HAZ at 24 months as a linear variable, the association with *Cryptosporidium* was further supported in an adjusted model (linear regression, *b* = -0.33, 95% CI -0.57, -0.08, *p* = 0.0009). Furthermore, there was additive impact of each additional infection on HAZ at 24 months (linear regression, *b* = -0.18, 95% CI -0.24, -0.026, *p* = 0.02).

**Table 2:** Adjusted odds ratio for *Cryptosporidium* infection among stunted 2-year old children. Polytomous logistic regression was used to assess for risk of *Cryptosporidium* infection by level of linear growth faltering at 24 months. Children were categorized by HAZ at 24 months: mild stunting HAZ < = -1 and > -2 (n = 111); moderate stunting HAZ < = -2 and >-3 (n = 131); severe stunting HAZ < = -3 (n = 72). Each group was tested against children with HAZ > -1 at 24 months (n = 51). Children with extreme stunting at birth (HAZ < -3.49) were excluded (n = 10). Confounding variables were included in the model.

Stunting at 24 months of age	Any *Cryptosporidium* infection (n = 302)		*Diarrheal Cryptosporidium infection (n = 100)*		Non-diarrheal Cryptosporidium infection (n = 202)	
	Odds ratio (95% CI)	*p*	Odds ratio (95% CI)	*p*	Odds ratio (95% CI)	*p*
**Severely Stunted** **HAZ ≤-3 (n = 72)**	2.69 (1.17, 6.15)	0.019	2.52 (0.91, 7.03)	0.076	2.78(1.15, 6.72)	0.023
**Moderately Stunted** **HAZ -2 to >-3 (n = 131)**	2.52 (1.23, 5.15)	0.011	2.76 (1.13, 6.76)	0.026	2.38 (1.10, 5.17	0.027
**Mildly Stunted** **HAZ -1 to >-2 (n = 111)**	2.27 (1.09, 4.71)	0.028	2.03 (0.80, 5.13)	0.135	2.39 (1.10, 5.26)	0.029

Among *Cryptosporidium spp*. infected children, the mean 24-month HAZ was significantly lower in children from households with lower monthly income (*t*-test, *p* = 0.0053). Among those from higher income households, *Cryptosporidium spp*. infected children had lower 24-month HAZ scores compared to uninfected children (*t*-test, *p* = 0.016). Additionally, there was no association between number of diarrheal episodes over the first 24 months of life and 24-month HAZ score (S1 Table).

## Discussion

In an urban slum in Bangladesh, we followed 392 children from birth to age two years of life. In this cohort, extreme poverty and malnutrition were common, affecting almost half of all households and half of enrolled children. Fifty-six percent of children met W.H.O criteria for moderate stunting at age two, which is higher than the reported rate of under-five stunting across South Asia (40.7%) [40]. Cryptosporidium spp. infection affected 77% of children in this cohort. Interestingly, we identified a larger number of asymptomatic infections than diarrheal infections. Potentially, this is due to consistent monthly surveillance sample collection and use of qPCR for diagnosis, rather than microscopy or antigen detection. In a birth cohort in India, Sarkar et al also reported a higher rate of asymptomatic than diarrheal Cryptosporidium infection [12].

In the current study, poverty was associated with *Cryptosporidium* infection, with children in households with more income less likely to have cryptosporidiosis. Based on gp-60 genotyping, we identified a predominance of *C*. *hominis* isolates in this cohort, which is consistent with other reports from the South Asian subcontinent [41–44]. Previously described risk factors for cryptosporidiosis in children include close contact with domesticated animals [20–22], crowded living conditions [12,25,26], and malnutrition [12,14]. We did not find the same association between domesticated animals and infection, likely because *C*. *hominis* predominated in our study population, and spread is anthroponotic. Additionally, we hypothesize that lower household income is related to overcrowding, and overcrowding is associated specifically with *C*. *hominis* infection [22]. Our study was not able to measure level of overcrowding in households, but median household size was 5, with a range of 2 to 18 persons per home, and this figure was not significantly different between groups. We did evaluate malnutrition and subsequent *Cryptosporidium* infection, however, there was no significant association. This was likely due to our study design that enrolled children from birth. We excluded children with extreme stunting at birth, as we were interested in controlling for peri-natal factors that may have led to stunting at birth and not potential maternal or prenatal factors. In non-birth cohort designed studies it would be difficult to differentiate between children stunted at birth and those who developed stunting perinatally.

One of the most significant findings of this study was the predisposition towards linear growth faltering that occurred in *Cryptosporidium spp* infected children. While malnutrition at birth did not predispose to *Cryptosporidium spp* infection, children who had at least one *Cryptosporidium spp* infection in the first two years of life had significantly worse nutritional status at 24 months, independent of income and maternal factors, suggesting that *Cryptosporidium spp* infection is associated with downstream growth faltering. Notably, both diarrheal and non-diarrheal infections were associated with subsequent stunting. This is supported by prior studies from Peru that have shown that children with asymptomatic and symptomatic *Cryptosporidium spp* infection had less weight gain in the first month of infection [32], but in contrast to the Peruvian studies, we found that children infected by *Cryptosporidium* even after 6 months of age, do not have “catch up growth” and once infected, are on a trajectory to growth stunting [45].

Our findings suggest that even a single *Cryptosporidium spp* infection at any point in the first two years of life, whether diarrheal or non-diarrheal, can be detrimental to a child’s physical development, resulting in impaired growth at age two. Therefore, we propose that malnutrition, rather than diarrhea, should be considered the most important outcome of *Cryptosporidium spp* infection in children. We have shown that non-diarrheal *Cryptosporidium spp* infection is widely prevalent in this cohort. The mechanism between non-diarrheal infection and malnutrition requires further study. *Cryptosporidium* infection has been associated with increased inflammation of the gut and loss of villus architecture [46] and murine models suggest that immune signaling in the gut may be disrupted resulting in enteropathy and poor growth [47].

Our study is limited in that we did not assess for multiple enteric pathogens. However, the aim of our study was to describe the total burden of *Cryptosporidium* infection in this cohort, rather than ascribe etiologies of diarrhea. Previous studies in this area have found that children may carry four or more pathogens in any given stool specimen [48]. It is possible that the presence of other enteric infections, or even other disease processes that were not evaluated in this study (e.g. acute respiratory infections) contribute to stunting. However, in this study we did not find a relationship between total burden of diarrhea and HAZ at 24 months. Therefore, we would argue that it is the presence of enteric infection, rather than the phenotype of diarrhea, that is contributing to stunting.

Based on our findings, future studies of cryptosporidiosis should aim to further study genotypic differences. We have demonstrated that in our cohort with *C*. *hominis* predominance, risk factors for infection are significantly different than in other populations with potentially different species. This may also impact means of transmission of infection. And beyond the species level, there may be additional clinical and immunologic differences between different subtypes of *C*. *hominis* that have not yet been described. Further studies of *Cryptosporidium spp*. genotyping will be important for informing strategies for prevention and treatment.

We have demonstrated that poverty, malnutrition, and *Cryptosporidium spp*. infection remain intricately connected. Worldwide, an estimated 178 million children under 5 suffer from stunted growth [40] and stunted growth in the first two years of life leads to irreversible damage, contributing to poor cognitive development, poor educational performance, and reduced earning potential in adulthood, trapping individuals in a lifetime of poverty [49,50]. Therefore, in populations like the Mirpur cohort, where cryptosporidiosis is found in 80% of children less than two years of age, tackling strategies for interrupting spread of infection, vaccination, and treatment, may have a staggering impact on human potential. Elimination of cryptosporidiosis may be one important step towards improving the condition of impoverished children around the world.

## Supporting Information

S1 Checklist STROBE Checklist. (DOC)

S1 Table Number of diarrheal episodes and HAZ scores at 24 months. (DOCX)
