# Supplementary material for: Natural History of Cryptosporidiosis in a Longitudinal Study of Slum-Dwelling Bangladeshi Children: Association with Severe Malnutrition
Source: PLoS Negl Trop Dis. 2016 May 4;10(5):e0004564. doi: 10.1371/journal.pntd.0004564 (PMC4856361; doi:10.1371/journal.pntd.0004564)
Supplement: S1 Table — (DOCX) [file pntd.0004564.s002.docx]

S1 Table. Number of diarrheal episodes and HAZ scores at 24 months. There was no significant association between a higher number of diarrheal episodes during the first two years of life and a lower HAZ score at 24 months.

| **Child’s infection status during 24 months** | ***n*** | **Number of diarrheal episodes during 24 months** | **mean HAZ**  **at 24 months** | **SD** | **T-test *p*-value** |
| --- | --- | --- | --- | --- | --- |
| Never had Crypto infection | 49 | <=4 | -1.9 | 1.159 | 0.56 |
|  | 27 | > 4 | -2.06 | 1.104 |  |
| Had Crypto positive diarrheal infection | 134 | <=4 | -2.31 | 1.27 | 0.56 |
|  | 120 | > 4 | -2.23 | 0.87 |  |
| Had Crypto positive asymptomatic infection | 32 | <=4 | -2.36 | 1.18 | 0.71 |
|  | 66 | > 4 | -2.28 | 0.92 |  |
